# Research on Fatigue Damage and Pitting Mechanism of Gears in Offshore Wind Power

**DOI:** 10.3390/ma19081505

**Published:** 2026-04-09

**Authors:** Zongchuang Zhu, Shiya He, Zhe Wang, Zhelun Ma

**Affiliations:** 1 School of Mechanical Engineering and Automation, Northeastern University, Shenyang 110819, China; 20235069@stu.neu.edu.cn (Z.Z.);; 2 Foshan Graduate School of Innovation, Northeastern University, Foshan 528312, China

**Keywords:** wind turbine gear, fatigue damage, pitting mechanism, finite element analysis

## Abstract

In response to the problem that the gears for offshore wind power are prone to cyclic stress and pitting damage under specific conditions, a finite element analysis method was adopted to establish numerical models for the distribution of cyclic stress on the gears and the dynamic expansion of pitting. Based on the material properties of ASTM5140 alloy structural steel, simulations were conducted using ANSYS 2024 R1 for contact stress analysis during gear meshing and COMSOL 6.3 for the evolution of pitting in a corrosive environment over a 120-h period. The results showed significant stress concentration in the tooth root fillet area under cyclic loads, with a maximum equivalent contact stress of 2.838 × 10^8^ Pa, which was identified as the key region for fatigue damage. Based on the simulated stress amplitude and material fatigue parameters, the predicted fatigue life of the gear under typical offshore operating conditions was approximately 13.3 years. In the corrosive environment, pitting pits exhibited an accelerating expansion trend, with pit volume increasing by approximately 125% and internal surface area by approximately 54% over 120 h. The volume growth followed a cubic polynomial, and the surface area growth followed a quadratic polynomial over time. These research results provide a quantitative basis for fatigue life assessment and corrosion protection design of offshore wind power gears.

## 1. Introduction

Currently, global issues such as environmental pollution, energy shortage, and the greenhouse effect are becoming increasingly severe, and promoting the development and utilization of sustainable energy has become a universal consensus in the international community [1]. As a typical and large-scale renewable energy source, offshore wind power can significantly reduce most environmental problems, such as air pollution caused by the use of traditional energy, represented by coal, from the source [2]. The planetary gearbox, as the core component for power transmission and speed regulation in offshore wind turbines, has a long service life and high reliability, which are crucial to ensuring the long-term stable operation of the unit [3]. However, offshore wind power equipment is long-term exposed to extreme environments composed of high humidity, high salt spray, and alternating loads, making gear components highly susceptible to fatigue damage caused by pitting and stress concentration [4,5,6]. This not only greatly shortens the service life of gears but also may bring high operation and maintenance costs and serious safety risks.

In recent years, surface modification and remanufacturing technologies such as laser cladding have been continuously developed, providing a feasible technical approach for in situ repair and performance restoration of wind power gears [7]. However, the effectiveness and reliability of repair processes fundamentally depend on the accurate analysis of the fatigue and corrosion mechanisms in the damaged area. Therefore, conducting research on the damage mechanism of gears under the combined action of alternating loads and corrosive media, and clarifying the expansion behavior of their fatigue damage and pitting, is a prerequisite for implementing targeted repair and service life extension.

At present, scholars at home and abroad have carried out relevant research on the fatigue characteristics of gears. Vitali et al. [8] investigated the fatigue strength of the planet carrier of wind turbine gearboxes through scale-down tests, verifying the applicability of small-scale specimens in fatigue testing. However, the study lacked detailed numerical simulation analysis of cyclic stress distribution, making it difficult to quantitatively describe the stress fluctuation characteristics of key areas such as root fillets. Liu et al. [9] addressed the problem of modeling the life correlation of wind turbine gearbox components by introducing the Copula function to construct a system reliability analysis model. Nevertheless, the model failed to conduct real-time analysis of the dynamic loads on wind turbine gearboxes under actual service conditions, thus being unable to accurately depict the evolution characteristics of the fatigue life of key gear parts under variable loads. Wang et al. [10] took a 5 MW wind turbine gearbox as the research object and proposed a fatigue damage prediction framework integrating environmental randomness and load time sequence. Combined with the 3D turbulent wind model and multi-body dynamics simulation, the study verified the applicability of the nonlinear damage correction model in predicting the fatigue damage of wind turbine gears. However, it did not incorporate the stress transfer effect of multiple components of the gearbox, leading to the inability to quantitatively describe the stress evolution of key areas such as root fillets and tooth contact spots.

Absolutely, relevant scholars have also conducted certain research on metal corrosion. Tsuyuki et al. [11] established a pH-dependent phase-field model for pitting corrosion of iron-based materials, revealing the anisotropic expansion behavior of corrosion pits. However, this model took pure iron as the research object and did not cover the corrosion response differences in low-alloy steel in high-salt environments. Jakubowski et al. [12] investigated the pitting corrosion mechanism of marine steels in the marine environment and verified the applicability of relevant models in pit depth prediction. However, the study did not conduct analysis for the specific working conditions of offshore wind turbine gearboxes, nor did it explore the influence of pitting in combination with gear structures, making it difficult to quantify the pitting failure law of key gear parts. Dey et al. [13] investigated the influence of pitting corrosion on the fatigue life of materials, clarified the differences in pitting susceptibility of materials under different temper conditions, and verified the applicability of image analysis technology in pitting characterization. However, the study did not conduct analysis for the working conditions of offshore wind turbine gearboxes, nor did it explore the pitting failure law in combination with gear structures, making it difficult to quantify the pitting-induced fatigue failure characteristics of key parts of wind turbine gears.

As an effective tool for analyzing the mechanical behavior and corrosion evolution of complex structures, the finite element method has been widely used in the field of mechanical structure failure prediction. However, for offshore wind power gears, existing studies mostly focus on the analysis of single factors such as cyclic stress or pitting corrosion, and there is a lack of parallel systematic investigation of these two failure mechanisms under extreme service environments. In particular, there is a lack of fine finite element models for gear stress response under dynamic loads and pitting expansion in corrosive media, which limits the comprehensive understanding of their respective damage characteristics.

To make up for the above research deficiencies, this paper adopts the finite element analysis method to establish corresponding numerical analysis models for the fatigue characteristics of offshore wind power gears under cyclic stress and the pitting expansion behavior in corrosive environments, respectively. Through the simulation of tooth surface contact stress distribution under cyclic loads and the simulation of the dynamic evolution process of pitting, the potential failure mechanisms of gears are revealed from the two dimensions of mechanical load and chemical corrosion. The research results can provide theoretical reference and technical basis for the fatigue strength design, corrosion protection, and life evaluation of offshore wind power gears.

## 2. Fatigue Damage Analysis of Wind Power Gears

### 2.1. Mechanism of Cyclic Stress on Meshing Gears

Cyclic stress refers to the general term for alternating contact stress and alternating bending stress borne by the tooth surface and tooth root during the periodic meshing of gears [14]. Therefore, cyclic stress is the core mechanical factor leading to gear failure, directly determining the service life and operational reliability of gears. The impact degree of cyclic stress on meshing gears is controlled by the coupling effect of four core factors: gear parameters, materials and processes, operating conditions, and lubrication conditions [15]. Each factor ultimately affects the fatigue failure mode and service life of gears by changing the stress amplitude, cycle characteristics, or stress concentration degree.

The damage mechanism of cyclic stress on gears is a process in which micro-defects of materials accumulate to macro-failure under the alternating stress field. The core mechanisms combined with quantitative formulas are as follows:

(1) Tooth surface contact fatigue mechanism. The tooth surface bears alternating Hertz contact stress:

(1) $$\sigma_{H}=Z_{H}Z_{E}Z_{\varepsilon }\sqrt{\frac{F_{t}}{bd_{1}}\cdot \frac{u\pm 1}{u}}$$

In the formula, $Z_{H}$ is the node area coefficient; $Z_{E}$ is the elastic coefficient; $Z_{\varepsilon }$ is the contact ratio coefficient; $F_{t}$ is the gear tangential force; b is the gear contact width; $d_{1}$ is the reference circle diameter of the pinion; u is the gear ratio.

Cyclic stress causes the peak value of subsurface shear stress of the gear to change periodically with the cycle, inducing cyclic plastic deformation. Dislocations accumulate to form microcracks, which expand along the direction of maximum shear stress. After lubricating oil seeps in, it is squeezed to accelerate the opening of cracks, and finally, the material falls off to form pitting. When this occurs $\sigma_{H}>H_{lim}$, the fatigue life satisfies:

(2) $$N_{f}=N_{0}{\left(\frac{\sigma_{Hlim}}{\sigma_{H}}\right)}^{m},(m=6∼10)$$

In the formula, $N_{0}$ is the test reference life; $\sigma_{\mathrm{Hlim}}$ is the contact fatigue limit of the gear material; $\sigma_{H}$ is the actual working contact stress of the tooth surface; m is the stress-life index.

Therefore, it can be known from the formula that the increase in stress amplitude will shorten the life exponentially.

(2) Tooth root bending fatigue mechanism. The dangerous section of the tooth root bears alternating bending stress:

(3) $$\sigma_{F}=\frac{F_{t}K_{A}K_{V}K_{F}Y_{Fa}Y_{Sa}}{bm}$$

In the formula, $F_{t}$ is the gear tangential force; $K_{A}$ is the application factor; $K_{V}$ is the dynamic load factor; $K_{F}$ is the load distribution factor between teeth; $Y_{\mathrm{Fa}}$ is the tooth profile factor; $Y_{\mathrm{Sa}}$ is the stress correction factor; b is the gear width; m is the gear module.

(3) Stress concentration effect of tooth root fillet:

(4) $$\sigma_{Fmax}=K_{f}\sigma_{Fnom}$$

In the formula, $K_{f}$ is the stress concentration coefficient of the tooth root; $\sigma_{\mathrm{Fnom}}$ is the nominal bending stress of the dangerous section of the tooth root.

This makes the local stress much higher than the nominal value. Under cyclic tensile and compressive loads, microcracks initiate at the defects. The cracks expand along the direction of maximum shear stress with stress cycles, and instantaneous fracture occurs when the depth reaches the critical value. Its life follows:

(5) $$N_{f}=N_{0}{\left(\frac{\sigma_{-1F}}{\sigma_{F}}\right)}^{n},(n=6∼15)$$

In the formula, $N_{0}$ is the reference cycle number; n is the fatigue index; $\sigma_{-1F}$ is the bending fatigue limit under symmetric cycle; $\sigma_{a}$ is the effective stress amplitude. The reduction in the stress concentration coefficient K_f_ can significantly extend the service life [16].

(4) Damage accumulation mechanism both types of damage comply with the Miner cumulative damage criterion:

(6) $$D=\sum \frac{N_{i}}{N_{fi}}$$

In the formula, $N_{i}$ is the number of stress cycles actually borne by the gear; $N_{\mathrm{fi}}$ is the corresponding gear fatigue life.

When D=1, the gear fails, reflecting the essential characteristic of cyclic stress that “micro-damage accumulates sequentially to cause macro-failure” [17].

### 2.2. Fatigue Damage Analysis Model of Meshing Gears

**Figure 1:**
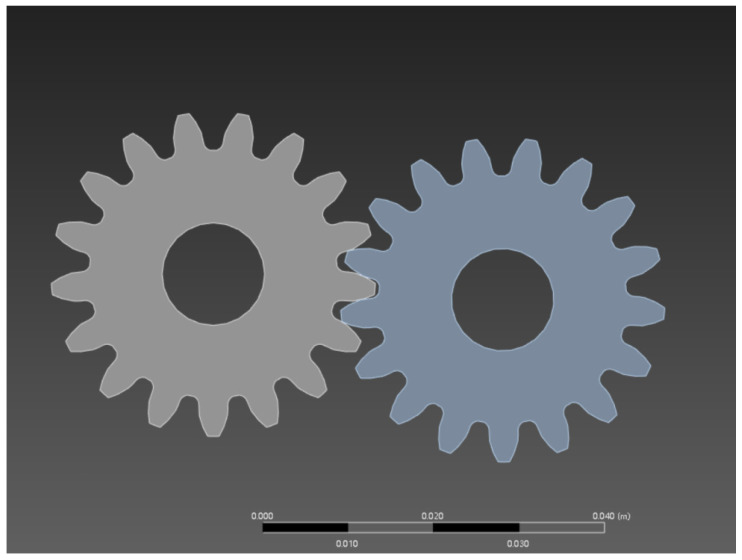
Meshing gear model.

ANSYS 2024 R1 is used for model simulation analysis, and two gears of the same model are used for modeling. Combined with the research background, to balance calculation efficiency, accuracy, and engineering representativeness, small-sized gears are selected as the research object for finite element analysis in this paper. The established geometric model is shown in Figure 1.

The specific gear parameters are shown in Table 1:

In this study, ASTM5140 (American Society for Testing and Materials Standard 5140 Steel) alloy structural steel is selected as the gear material, and the specific parameters are shown in Table 2.

### 2.3. Model Setting and Solution

**Figure 2:**
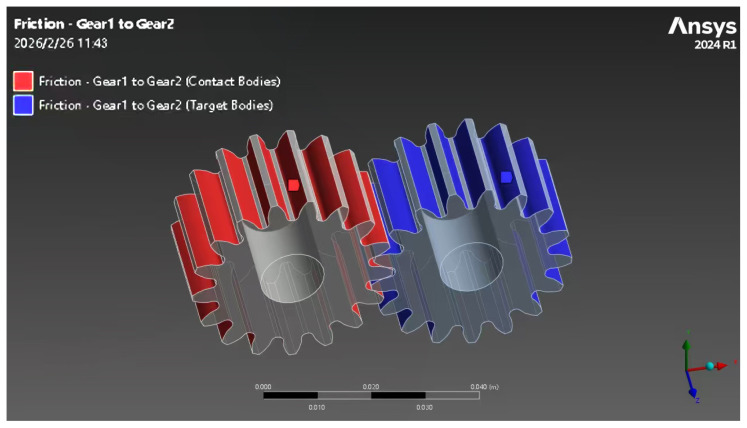
Gear contact surface.

The tooth surfaces of the two gears are set as contact pairs, and the contact type is defined as frictional contact. Based on the properties of ASTM5140 alloy steel (quenched and tempered hardness of 220–280 HB, poor seawater lubricity) and the measured range of 0.12–0.18, a typical compromise value of 0.15 is adopted as the friction coefficient. The gear contact surface setting is shown in Figure 2.

The advanced settings of the contact surface are as follows:

(1) Normal stiffness setting: Normal stiffness refers to the ability of the contact interface to resist elastic deformation in the direction perpendicular to the contact surface, representing the normal load required for the contact pair to produce unit normal deformation [18]. In this study, the normal stiffness is set to “Factor” with a value of 1. This matches the stiffness characteristics of the gear material constitutive model through the proportional coefficient, simplifying parameter input while ensuring the rationality of contact stiffness.
(2) Stiffness update setting: Stiffness update is an operation in contact analysis that dynamically adjusts the contact stiffness matrix according to the contact state (such as contact area and load distribution) in each iteration. In the simulation of gear cyclic meshing, the gear contact area and load change dynamically with the meshing position. In this study, the stiffness update is set to “Every Iteration” to adapt to the dynamic changes of the contact state during gear cyclic meshing and improve the accuracy of stress calculation.
(3) Time step setting: Time step is a time unit in numerical simulation that discretizes the continuous physical process into multiple time intervals for step-by-step calculation. In this study, the time step is set to “Automatic Division”, which balances the calculation efficiency and convergence stability under cyclic loads through adaptive step size control.

**Figure 3:**
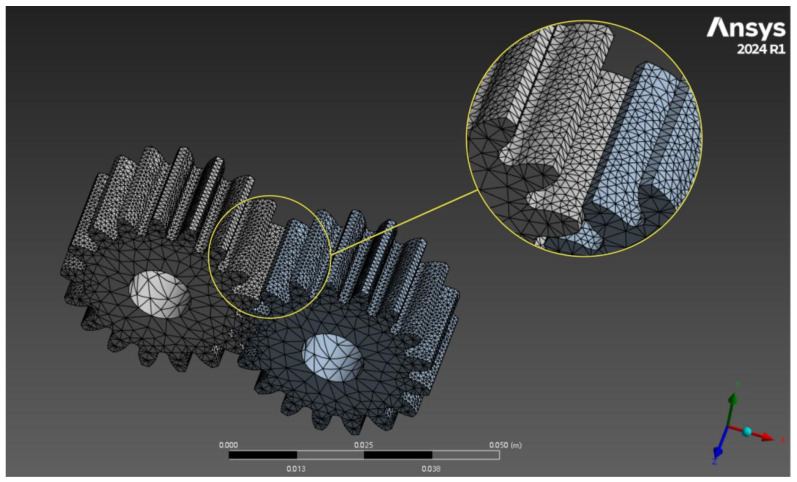
Gear meshing division.

In this study, tetrahedral meshes are used for model discretization, which can realize high-precision mesh filling of the entire model while ensuring the efficiency and accuracy of gear mesh generation. At the same time, to improve the calculation accuracy, the meshes of the contact surfaces of the two gears are refined, with the element size set to 0.5 mm, and the rest are kept as conventional. The gear mesh generation is shown in Figure 3.

**Figure 4:**
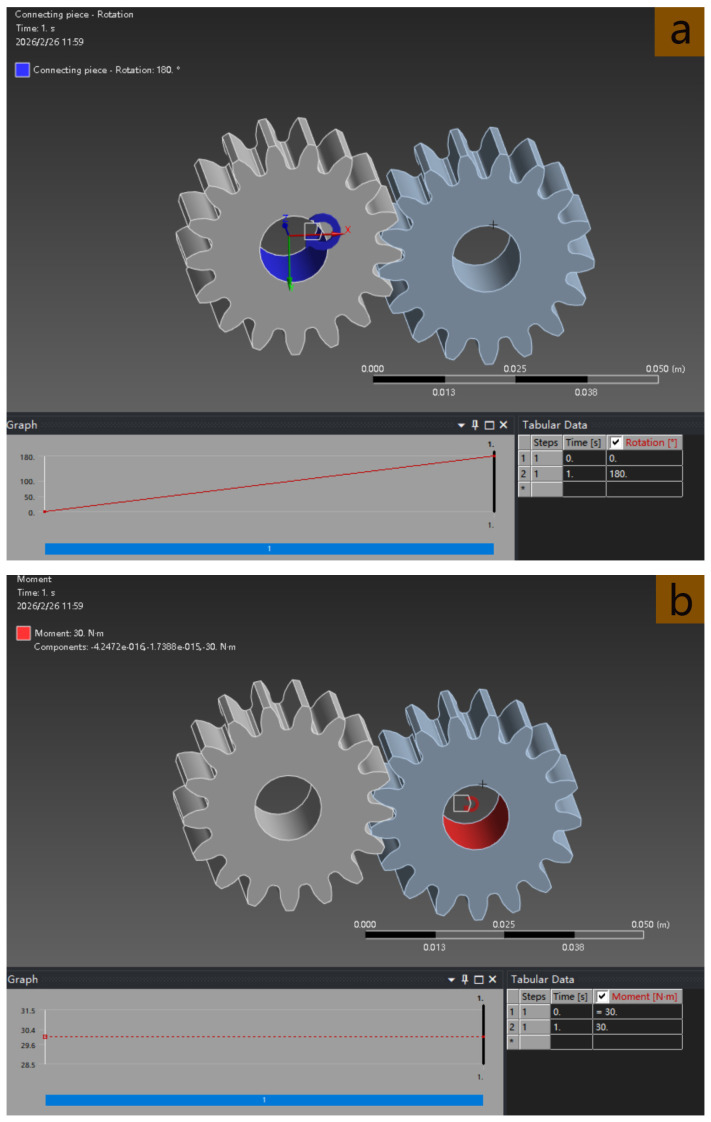
Gear meshing setting. (**a**)—Rotation setting of the driving gear; (**b**)—Torque setting of the driven gear.

To simulate the power transmission characteristics under actual load conditions, the driving gear is set to rotate at an angle of 180°/min, and the driven gear is set to a torque of 30 N·m to realize meshing rotation and stress measurement. The rotation setting of the driving gear and the torque setting of the driven gear are shown in Figure 4.

### 2.4. Result Analysis

**Figure 5:**
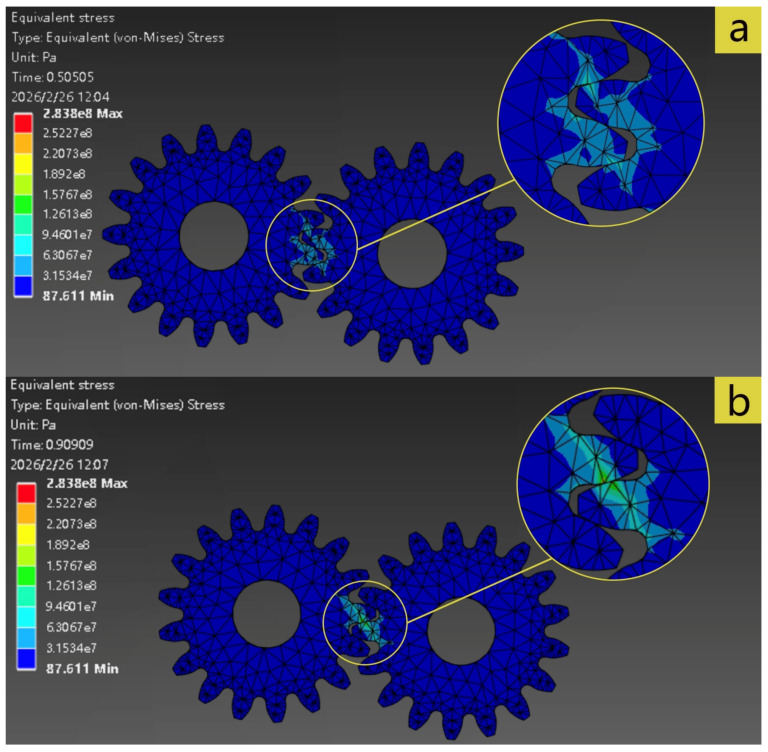
Cloud diagram of contact stress distribution. (**a**)—Frame 1; (**b**)—Frame 2.

Based on the finite element simulation results, this section focuses on the gear contact stress distribution characteristics and fatigue life prediction. Two frames of stress distribution results are randomly intercepted during the meshing process, as shown in Figure 5.

It can be seen from Figure 5 that there is obvious stress concentration in the tooth root area, and the spatial maximum equivalent contact stress appears at the tooth root fillet, with a value of approximately 2.838 × 10^8^ Pa, which is significantly higher than other areas of the tooth surface. The spatial maximum stress occurs in the tooth root fillet region, which is essentially generated by the gear tooth functioning as a “cantilever beam” subjected to bending loads, and is sharply amplified due to the geometric discontinuity. Therefore, to verify the correctness of the finite element analysis results, bending stress theory is employed for validation. For Equation (3), based on the data in Table 1 and Table 2, we can calculate $F_{t}=\frac{2T}{d_{1}}=\frac{2\times 30,000}{34}=1764.7\;N$. Considering that the gearbox operates under steady loads without severe impacts, the application factor is taken as $K_{A}=1.0$. Based on the gear accuracy and pitch line velocity, the gear accuracy is estimated to be Grade 7, and the pitch line velocity is relatively low, so the dynamic factor can be taken as $K_{V}=1.0$, meaning the influence of dynamic loads is neglected. For standard spur gears, near the highest point of single-tooth contact, the load is fully borne by a single tooth pair; therefore, $K_{F}=1.0$. Even considering the influence of multi-tooth meshing, according to the contact ratio $\varepsilon_{a}=1.516$, the load might vary slightly outside the single-tooth contact zone, but for a conservative calculation, $K_{F}=1.0$ is adopted. According to the Mechanical Design Manual, when z=17, $Y_{Fa}\approx 2.97,\;Y_{Sa}\approx 1.53$. Substituting into Equation (3) yields $\sigma_{F}=2.0046\times 10^{8}\;Pa$. In actual operating conditions, there may be slight impacts, dynamic loads, and non-uniform distributions, and the factors $K_{A}$, $K_{V}$ and $K_{F}$ are typically greater than 1. Nevertheless, the finite element results and the theoretical values are consistent in order of magnitude, indicating the rationality of the finite element model.

Comparing the stress distribution nephograms at different times, it can be seen that the high-stress area shifts with the rotation of the gear, indicating that the load transmission during the meshing process has dynamic characteristics. The contact stress reaches the peak during the single-tooth meshing stage and tends to be evenly distributed after entering the double-tooth meshing. However, stress concentration always exists at the tooth root throughout the process. The reason is that the sudden change in the geometric shape of this position causes the stress flow lines to be densely distributed here, forming a local high-stress area, which easily induces micro-plastic accumulation and crack initiation, thus becoming the starting position of fatigue damage. At the same time, the stress concentration area of the tooth root is consistent with the position of the dangerous section in the bending fatigue theory, indicating that the simulation results effectively support the prediction and evaluation of gear fatigue life.

It is worth noting that from the color gradient of the nephogram, the contact stress near the tooth root decreases rapidly from 2.838 × 10^8^ Pa to 1.892 × 10^8^ Pa and below, with a significant stress gradient. The change in the gradient is likely to lead to the accumulation of local plastic deformation, and coupled with cyclic loads, it is very easy to induce the initiation of microcracks. In addition, the uneven distribution of contact stress on the tooth surface may aggravate surface fatigue and pitting damage.

**Figure 6:**
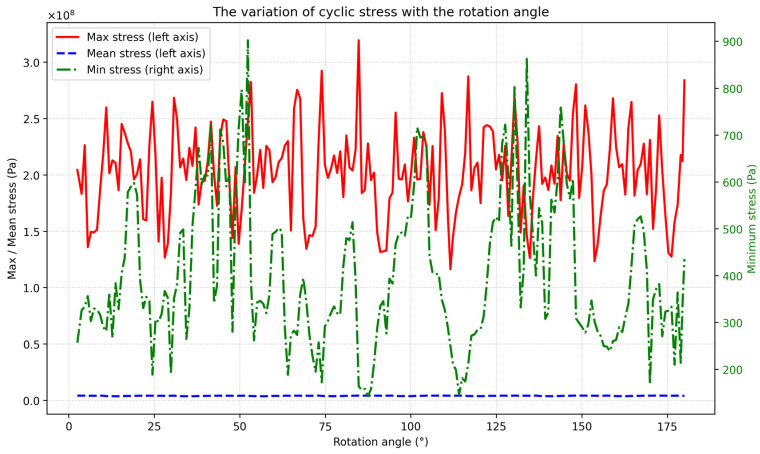
Contact stress of intermeshing gears.

The specific contact stress data are exported and visualized, as shown in Figure 6:

It can be seen from the visualization results that the maximum stress fluctuates periodically with the gear rotation in synchronization with the meshing cycle, and the maximum stress in the stress cycle is concentrated in the range of 2.5 × 10^8^ Pa–3.0 × 10^8^ Pa; the minimum stress in the stress cycle is always in the range of 150–900 Pa, which is too different from the maximum stress and can be ignored in engineering analysis. It is worth noting that although the maximum stress fluctuates significantly, the mean stress in the stress cycle remains stable in the range of 0.03 × 10^8^ Pa–0.05 × 10^8^ Pa, indicating that this area bears alternating bending loads with approximately symmetric cycles.

The fitting formula of the S–N (Stress–Life) curve in the high-stress region conforms to Formula (5). According to the cyclic stress simulation results, the maximum cyclic stress $\sigma_{max}$ is 3.2 × 10^8^ Pa, and the minimum stress $\sigma_{min}$ is ignored because its value is much lower than the maximum stress, so the effective stress amplitude $\sigma_{a}$ is taken as 160 MPa; for ASTM5140 material, $\sigma_{-1}$ = 350–400 MPa, and 375 MPa is adopted; n is 9, and N_0_ is 10^6^ cycles. At the same time, considering the actual working conditions, the working condition correction coefficient K is introduced, with a value of 0.6 [19]. Substituting the above data into the formula, N_f_ = 1.28 × 10^9^ cycles is obtained. Combined with the actual operation parameters of the offshore wind turbine, the input speed of the gearbox is 200 r/min, and the annual operation time is 8000 h, so the actual service life is calculated to be approximately 13.3 years.

## 3. Pitting Mechanism of Wind Power Gears

### 3.1. Pitting Mechanism

Pitting, also known as pitting corrosion, is a corrosion form that concentrates in a small range on the metal surface and penetrates into the metal interior. It is a local corrosion phenomenon in which corrosion pits form on the initially smooth metal surface. Research on pitting of traditional gears is relatively mature, and existing studies focus on pitting problems in special application scenarios [20]. Due to the special service environment of wind power gears, microcracks appear after fatigue damage, and pitting occurs rapidly at the microcracks in the salt spray environment, accelerating the wear of wind power gears. On the other hand, the expansion of corrosion pits is affected by many factors, such as pH value, material type, temperature, and humidity, making research on the gear pitting mechanism in this environment relatively complex. Therefore, in this study, the pitting mechanism is simplified as follows:

On the metal surface outside the corrosion pit, the reduction reaction of oxygen occurs, as shown in Formula (7).

(7) $$4H^{+}+4e^{-}+O_{2}\to 2H_{2}O$$

Inside the corrosion pit, the oxidation reaction of the metal occurs, taking metallic iron as an example here.

(8) $$Fe(s)⟶{Fe}^{2+}+2e^{-}$$

The dissolved iron reacts with hydroxide ions in the electrolyte on the electrode surface to form iron hydroxide precipitation.

(9) $$H^{+}+{OH}^{-}⟶H_{2}O$$

At the same time, the production of iron hydroxide also causes the ionization of water to form protons to balance the consumed hydroxide ions.

(10) $$H_{2}O⇌H^{+}+{OH}^{-}$$

It is worth noting that the consumption of hydroxide ions in Formula (9) leads to a decrease in the pH value on the electrode surface, thereby accelerating the metal dissolution inside the corrosion pit.

### 3.2. Pitting Model Construction

Before conducting finite element analysis, it is necessary to determine the material type of the gear model. The material used in this study is ASTM5140 alloy structural steel.

**Figure 7:**
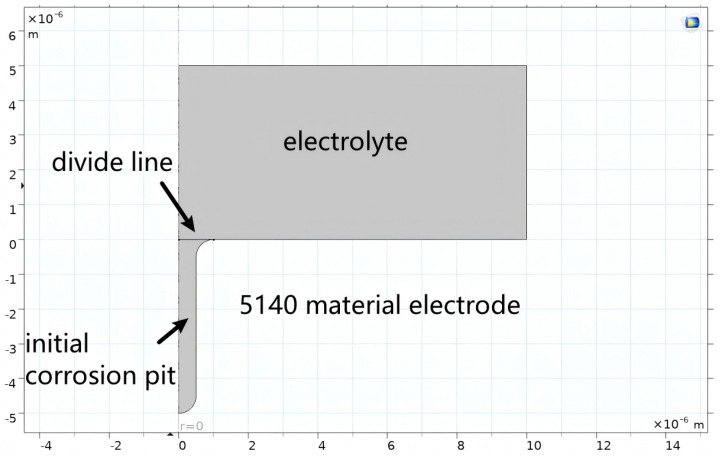
Initial geometric model.

To simplify the model structure, a two-dimensional axisymmetric geometric model was constructed in this study, as shown in Figure 7. The software used is COMSOL 6.3.

### 3.3. Model Setting and Solution

According to the offshore working conditions of the equipment, assuming that the salt concentration inside the pit is consistent with the seawater composition, the following parameter values are defined, as shown in Table 3.

The porosity represents the volume fraction of electrolyte within the pit, accounting for the porous layer formed by corrosion products. In this experiment, tertiary current distribution is adopted, the Nernst–Planck interface is used to define the model, and the charge conservation model is selected as aqueous phase electroneutrality. Fixed concentration and electrolyte phase potential are set on the top horizontal electrolyte boundary opposite to the bulk electrolyte, as detailed in Table 4.

Among them, the Na^+^ concentration is obtained according to the salt concentration, and the Fe^2+^ concentration and Cl^−^ concentration are calculated by Formulas (11) and (12). And Eeq_Fe represents the standard equilibrium electrode potential of iron, which is given relative to the SHE.

(11) $$c({Fe}^{2+})={\left[\frac{K_{eq\_Fe(OH{)}_{2}}}{c({OH}^{-})}\right]}^{2}$$

In the formula, Keq_Fe(OH)_2_ is the equilibrium constant.

(12) $$c({Cl}^{-})=c({Na}^{+})+c(H^{+})+2\times c({Fe}^{2+})-c({OH}^{-})$$

**Figure 8:**
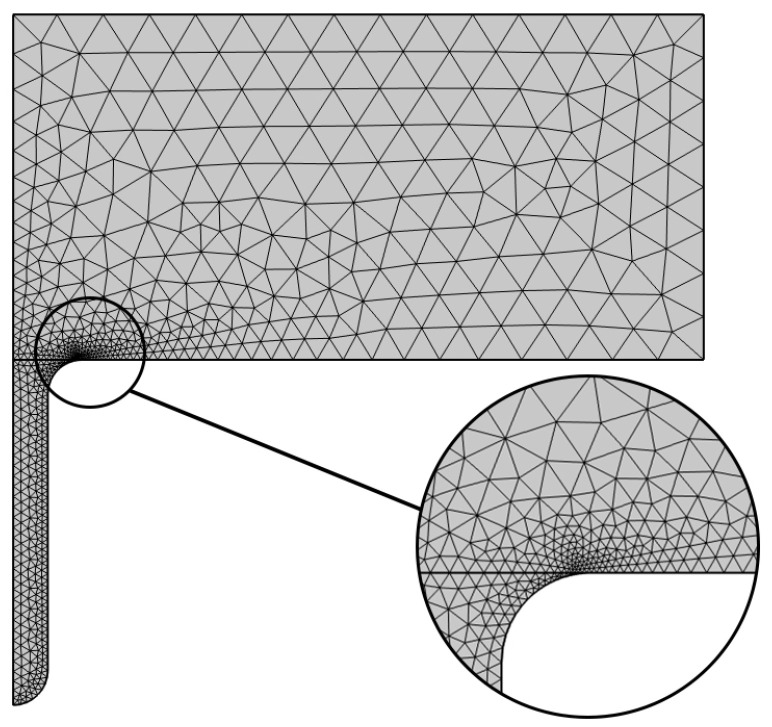
Grid division model diagram.

To obtain high-quality mesh generation results while shortening the solution time as much as possible, only the mesh of the corrosion pit part is set to “Extra Fine”, and the rest are set to “Normal”, as shown in Figure 8.

A transient solver is used to solve the model, and the volume and internal surface area of the corrosion pit are integrated to simulate the expansion of the corrosion pit within 120 h, with results output every 5 h.

### 3.4. Result Analysis

**Figure 9:**
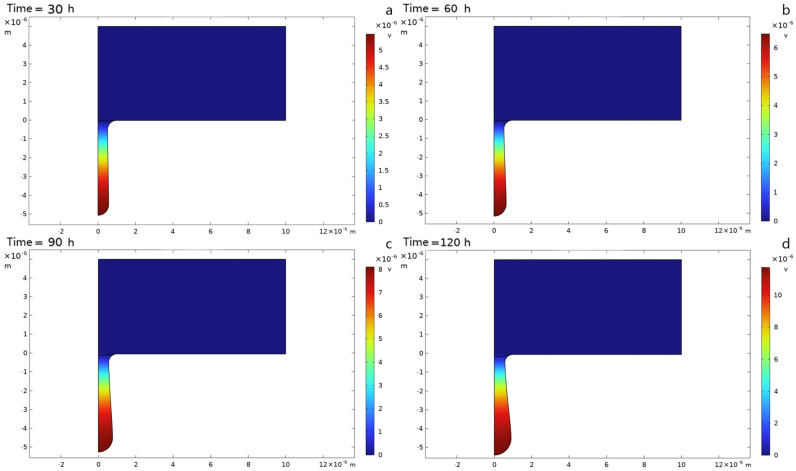
Potential of the corroding metal after 30 h, 60 h, 90 h and 120 h. (**a**) After 30 h (**b**) After 60 h (**c**) After 90 h (**d**) After 120 h.

The results obtained by the solver are exported, and the iron dissolution current density is analyzed, as shown in Figure 9.

In the figure, the surface represents the potential of the corroding metal, with the unit being V. It can be seen that the current density inside the corrosion pit is significantly higher than that in the area outside the pit, and an obvious convex corrosion pit forms under the metal surface. This indicates that in the offshore environment, the interior of the corrosion pit is the active area of the electrochemical reaction, and the peak value of the current density is located at the bottom of the corrosion pit, forming a significant gradient with the edge of the pit mouth. This distribution conforms to the “autocatalytic effect” of pitting, which further accelerates the dissolution of the metal and forms a positive feedback.

**Figure 10:**
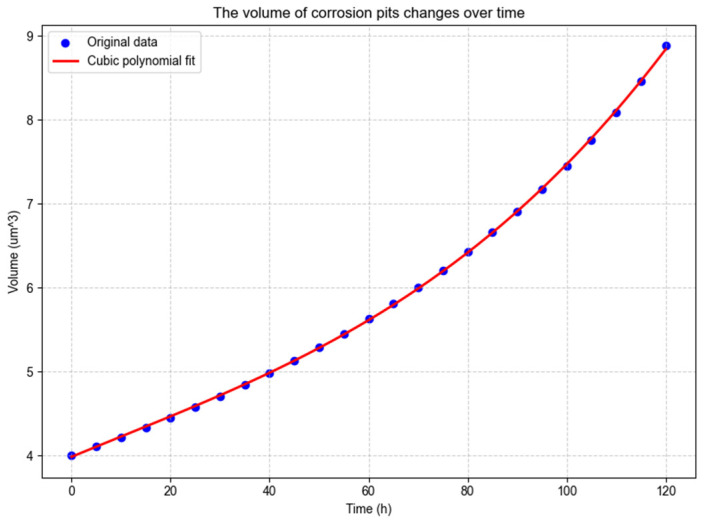
Variation in corrosion pit volume over time.

**Figure 11:**
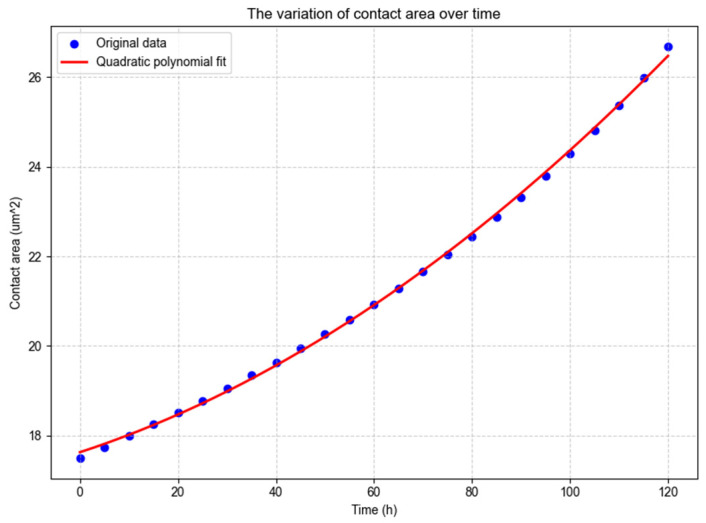
Changes in the surface area within the corrosion pits over time.

To further elucidate the pitting corrosion mechanism and the spatio-temporal evolution law of corrosion pit expansion, data fitting of corrosion pit volume and internal surface area was performed using Python 3.14.0 in PyCharm 2025.2.4 for qualitative analysis, as illustrated in Figure 10 and Figure 11.

The R^2^ reaches 0.99991, and the fitting expression is given according to the 95% confidence interval:

(13) $$V\;=\;1.4630\;\times \;10^{-6}t^{3}\;-\;4.1226\;\times \;10^{-5}t^{2}\;+\;0.0244t\;+\;3.9831$$

In the formula, V is the volume of the corrosion pit; t is the reaction time.

It can be seen from the figure and the expression that with the increase in time, the volume of the corrosion pit shows an accelerated growth trend, which is manifested by the gradual increase in the curve slope, increasing from the initial value of about 4.0 µm^3^ to more than 9.0 µm^3^ at the 120-h node. Corresponding to the pitting expansion process, the corrosion pit enters the “rapid expansion” stage from the “slow initiation” stage, which conforms to the mechanism that the corrosion rate increases exponentially with time in a high-salt environment. The R^2^ is close to 1, indicating that the cubic polynomial has an extremely high degree of fit with the actual data and can accurately predict the volume of the corrosion pit at different time nodes.

The R^2^ reaches 0.99925, and the fitting expression is given according to the 95% confidence interval:

(14) $$S\;=\;0.0003t^{2}\;+\;0.0358t\;+\;17.6261$$

In the formula, S is the surface area within the corrosion pits; t is the reaction time.

The internal surface area of the corrosion pit shows a continuous growth trend with time (the curve slope increases stably), increasing from the initial value of about 17.5 µm^2^ to more than 27.0 µm^2^ at the 120-h node. The growth of the internal surface area reflects the continuous expansion of the corrosion pit boundary, which is a direct manifestation of the synergistic effect of metal dissolution and ion migration; the quadratic polynomial fitting effectively describes this “stable acceleration” expansion law. The R^2^ reaches 0.99925, and the error (RMSE = 0.0786) is within a reasonable range, indicating that the fitting model reliably reflects the change law of the internal surface area.

**Figure 12:**
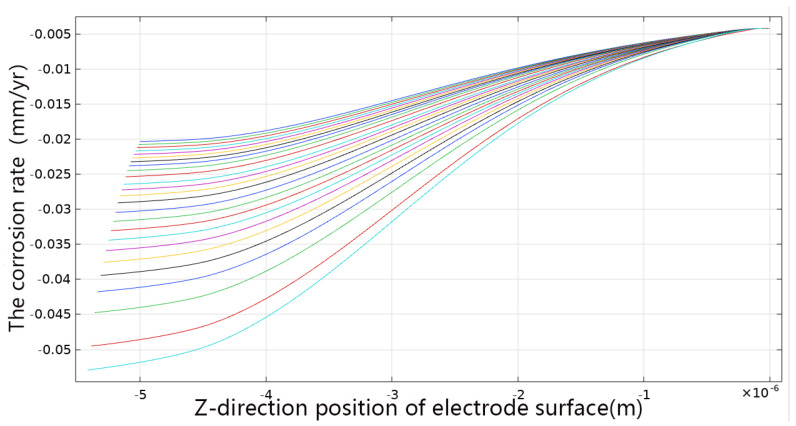
Electrode dissolution rate at different times.

In addition, the finite element software is used for the visualization of the corrosion rate for post-processing, as shown in Figure 12.

Figure 12 shows the corrosion rate distribution along the electrode surface at each 5-h interval. The top curve represents the initial state, and the bottom curve represents the final state. From top to bottom, they are the corresponding curves at intervals of 5 h. It can be seen that with the passage of time, the dissolution rate inside the corrosion pit increases significantly, and the peak rate area gradually concentrates at the bottom of the pit. This trend is consistent with the fitting results of volume and internal surface area, further verifying that pitting has obvious time-varying acceleration characteristics in a high-salt environment. In addition, the corrosion rate also increases to a certain extent at the edge of the pit, indicating that the corrosion pit is accompanied by edge activation and micro-pit merging during the expansion process.

It should be noted that although pitting corrosion macroscopically manifests as a chaotic phenomenon with random distribution and autocatalytic characteristics, its propagation process is essentially controlled by ion migration and diffusion at the microscopic scale, which is precisely what the Nernst–Planck equation accurately describes. In this study, the equation is explicitly coupled with nonlinear Butler-Volmer kinetics, where the iron dissolution rate is proportional to the local H^+^ concentration, thereby introducing the necessary heterogeneity and successfully capturing the acidification-driven autocatalytic effect at the pit bottom. Combined with the porous medium assumption (porosity 2.5%), as well as transient solving and geometric deformation, the model effectively describes the non-uniform mass transport and electrochemical coupling during pitting propagation, and the simulation results successfully reproduce local corrosion characteristics such as current density concentration at the pit bottom. Therefore, when constrained by appropriate electrochemical boundary conditions, the Nernst–Planck equation is not limited to uniform systems but can effectively quantify the non-uniform evolution of pitting corrosion.

Meanwhile, the current model simplifies the pitting initiation process: the initial pit geometry is predefined, and the dynamic formation and rupture of the passive film on the ASTM5140 steel surface are not explicitly considered. In fact, the passive film plays a key role in pitting control through the competitive mechanism of local rupture and repassivation, affecting ion diffusion and pit evolution. Future research will introduce a passive film evolution model, coupling current density with film coverage, to more realistically simulate the competitive mechanism of film rupture and repair during pitting initiation and propagation.

## 4. Conclusions

This paper employs the finite element method to investigate the fatigue damage and pitting process that the offshore wind turbine gears undergo under specific working conditions. Through the established simulation models, the study quantitatively reveals the key failure mechanisms from the two dimensions of mechanical load and electrochemical corrosion.

(1) The finite element analysis of gear meshing shows a significant stress concentration phenomenon in the tooth root fillet area during the periodic meshing process, with a maximum contact stress of 2.838 × 10^8^ Pa, which is about 1.5–2 times that of other areas. This region is identified as the most vulnerable site for fatigue crack initiation. Based on the simulated stress amplitude and material fatigue parameters, the fatigue life of the modeled gear under typical offshore operating conditions is predicted to be approximately 13.3 years, offering a quantitative basis for gear life evaluation and maintenance planning.
(2) The pitting corrosion model demonstrates that in a high-salinity offshore environment, corrosion pits exhibit an accelerated expansion trend. Within 120 h, the pit volume increases by about 125%, and the internal surface area increases by about 54%. The evolution follows distinct temporal laws—a cubic polynomial for volume growth and a quadratic polynomial for surface area growth—with high fitting accuracy (R^2^ > 0.999). This indicates that the corrosion rate intensifies over time, a critical factor for accurate residual life prediction in corrosive marine atmospheres.

This study provides theoretical guidance for predicting the service life of offshore wind power gears and offers valuable references for their design. However, certain limitations exist. The current model is based on a simplified gear configuration, and cyclic stress and corrosion are analyzed separately without incorporating their coupled effects. Future research will focus on developing a complete planetary gear system model to enable the analysis of the synergistic effects of stress and corrosion, thereby enhancing the engineering applicability of the model.

## Figures and Tables

**Table 1:** Parameters of gear model.

Module (mm)	Tooth Thickness (mm)	Number of Teeth	Pitch Diameter (mm)	Inner Diameter (mm)
2	20	17	34	12

**Table 2:** Properties of ASTM5140 material.

Density (kg·m^−3^)	Tensile Strength (MPa)	Yield Strength (MPa)	Hardness (HB)
7850	980	785	207

**Table 3:** Parameters of pitting corrosion model.

Salinity (mol·m^−3^)	Porosity	pH	Temperature (K)
600	0.025	10	293.15

**Table 4:** Concentration and electrolyte phase potential values.

Na^+^ Density (mol·m^−3^)	Cl^−^ Density (mol·m^−3^)	Fe^2+^ Density (mol·m^−3^)	Eeq_Fe (V)
600	599.93	9.6945 × 10^−5^	−0.44

## Data Availability

The original contributions presented in this study are included in the article. Further inquiries can be directed to the corresponding author.
